# Automatic Recognition of Road Damage Based on Lightweight Attentional Convolutional Neural Network

**DOI:** 10.3390/s22249599

**Published:** 2022-12-07

**Authors:** Han Liang, Seong-Cheol Lee, Suyoung Seo

**Affiliations:** Department of Civil Engineering, Kyungpook National University, Daegu 37224, Republic of Korea

**Keywords:** object detection, lightweight network, attention mechanism, road damage, computer vision

## Abstract

An efficient road damage detection system can reduce the risk of road defects to motorists and road maintenance costs to traffic management authorities, for which a lightweight end-to-end road damage detection network is proposed in this paper, aiming at fast and automatic accurate identification and classification of multiple types of road damage. The proposed technique consists of a backbone network based on a combination of lightweight feature detection modules constituted with a multi-scale feature fusion network, which is more beneficial for target identification and classification at different distances and angles than other studies. An embedded lightweight attention module was also developed that can enhance feature information by assigning weights to multi-scale convolutional kernels to improve detection accuracy with fewer parameters. The proposed model generally has higher performance and fewer parameters than other representative models. According to our practice tests, it can identify many types of road damage based on the images captured by vehicle cameras and meet the real-time detection required when piggybacking on mobile systems.

## 1. Introduction

Pavement damage due to road aging, traffic volume, construction materials, and weather [1,2] is an important cause of driving safety [3,4,5]. Therefore, pavement damage detection is beneficial for drivers’ lives’ safety. In addition, road infrastructure is a vital national asset, and understanding its damage level is crucial for its subsequent maintenance [6]. Moreover, road damage detection technology plays a crucial role in the construction of intelligent transportation systems (ITS) and automated assisted driving systems (ADAS) [7,8].

Early road damage inspection relied on manual progress along the road by walking or slow-moving vehicles and visually inspecting the road surface. Inspection results were highly subjective and time-consuming. In addition, this inspection task needed to be performed at slow speeds in the lane, and there was also the potential for traffic hazards for the staff [9].

Subsequently, some organizations use sensor-equipped inspection vehicles to collect pavement condition data, where expensive equipment such as laser scanning cameras, road profilers, and 3D capture cameras are required, which undoubtedly increases the cost of such systems significantly [10,11,12,13]. The collected pavement data need to be subsequently processed in the workstation, which is still very time-consuming [14,15].

With the development of deep learning techniques, many researchers have started using neural network-based models for road damage detection. Most of these works use convolutional neural networks (CNNs) for pixel-level segmentation of road images. For example, Fan et al. [16] first used a CNN-based classification network to filter images containing cracks, after which the damages were extracted by traditional image processing methods of filtering with adaptive thresholding. On the other hand, Feng et al. [17] pre-processed the images to filter image noise, input them into two different crack segmentation models, and finally used the predicted results to synthesize the geometric parameters of the cracks calculated using the prediction results. Subsequently, Nguyen et al. [18] proposed a two-stage CNN network for low-resolution image detection and segmentation, which shortens the processing steps while increasing the efficiency of automated detection. Cheng et al. [19] proposed a computerized road crack detection method based on the structure of U-Net and introduced a function of distance transformation to assign pixel weights according to the actual segmentation minimum distance to assign pixel weights. Rill-García et al. [20], on the other hand, used VGG19 to replace the original backbone feature extraction network (VGG16) based on U-Net for improving the accuracy of road crack segmentation in the presence of incorrect annotations.

However, the above methods have certain limitations, which exist in three main areas.

- Most pavement damage detection efforts obtain crack results by semantic segmentation of pixel-level images, which requires input images that must be high-quality images that closely match the pavement, undoubtedly increasing the cost and reducing the efficiency during initial image acquisition and making it difficult to meet the real-time warning required by ADAS.
- Although the state-of-the-artwork allows pixel-level segmentation of pavement cracks or potholes, no other pavement damage classification was considered. We believe that identifying specific pavement damage types, such as longitudinal or transverse cracks, alligator cracks, and potholes, is essential when performing road damage detection.
- Most related work cannot be automated end-to-end or lightweight model network construction due to the need for multi-stage operations, such as image pre-processing or post-processing.

Therefore, applying these research works to practical scenarios is very difficult considering these limitations. To address these issues, in this paper, we propose a lightweight end-to-end road damage detection network with the following main contributions:

1. We designed a backbone feature extraction network using a combination of lightweight feature detection modules to ensure efficient automatic feature extraction while making the model parameters smaller.
2. Our proposed multi-scale fusion network enriches the diversity of road damage features, improves the detection robustness of the algorithm at different scales, and facilitates detection efficiency when the distance and viewpoint change.
3. We propose a lightweight multi-branch channel attention network (LMCA-Net) for the road damage detection task. This embedded attention module can enhance feature information by assigning weights to multi-scale convolutional kernels depending on the object size, aiming to improve detection accuracy with smaller parameters.

Compared to other representative models, our proposed model generally performs better and has fewer parameters. Based on the images captured by vehicle cameras, it can also identify many types of road damage and meet the real-time detection requirements of mobile systems.

## 2. Related Work

### 2.1. Road Damage Detection Methods Based on Traditional Image Processing

Koch and Brilakis [21] proposed a method to automatically detect pavement potholes using histogram thresholding to segment the pavement damage region and elliptical regression of geometric features to determine the ROI region. Schiopu et al. [22] proposed a pothole detection that can eliminate false detection due to shadows of roadside objects, specifically in the ROI region, by setting a threshold value for the geometric features of potholes and presuming the potholes through a decision tree labelling.

Besides geometric morphology, some studies perform road damage detection from the perspective of image colour. For example, Jakštys et al. [23] outlined the edge contours of road potholes by analysing the B-component in the RGB colour space of road potholes in the ROI region. Akagic et al. [24], on the other hand, analysed the B-component in the RGB colour space by component to perform image segmentation of the asphalt pavement and then detected the pothole areas by processing methods such as cropping, Otsu thresholding, and boundary elimination.

Classical image processing to detect objects tends to segment the object from the background using thresholding, and most prior studies on road damage detection do the same. For example, Akagic et al. [25] proposed a pavement crack detection method based on a combination of the grayscale histogram and Otsu thresholding to search for pavement cracks by dividing the input image into sub-images after the ratio of the maximum histogram to the threshold value obtained. Sari et al. [26] brought results with reasonable accuracy by using the Otsu thresholding algorithm and Gray Level Co-occurrence Matrices (GLCM) for road crack feature detection and extraction, followed by the support vector machine (SVM) algorithm for experimental classification statistics.

Quan et al. [27] proposed an improved Otsu thresholding-based crack detection method that avoids the problem of peak prominence by modifying the weight factor and improves the accuracy compared to the original Otsu thresholding. Chung et al. [28] proposed a method to find the optimal threshold of the image using inverse binary and Otsu thresholding algorithm to meet the real-time pavement pothole detection. They applied the distance transformation of the image using the Watershed algorithm for calculating marker potholes.

In addition, many studies used the boundary decision capability of SVM to classify road damage. For example, Hoang [29] used the least squares version of SVM (LS-SVM) for supervised learning to establish an automatic classification method for pavement potholes compared to single pavement pothole detection. Gao et al. [30] used a machine learning model based on the library of support vector machines (LIBSVM) to propose a fast detection method that distinguishes potholes, longitudinal cracks, transverse cracks, and complex cracks.

These classical image processing methods have performed very well in the past, as shown in Table 1, with the advantage of not requiring large datasets for manual annotation. However, there are some unavoidable problems, as most of the above methods employ techniques such as colour segmentation, threshold feature detection, SVM, etc., which are limited by illumination variations, occlusions, colour variations, and complex backgrounds. Moreover, the need to design feature algorithms leads to a single type of detected road damage.

### 2.2. Deep Learning-Based Road Damage Detection Methods

With the rapid development of deep learning and artificial intelligence, the CNN has become the mainstream technology for road damage detection. The detection methods are mainly divided into image classification, semantic segmentation, and object detection.

Image classification: The most typical CNN approaches to perform road damage detection and classification tasks are usually trained by designing a neural network consisting of convolutional and fully connected (FC) layers. For example, An et al. [31] classified images into two types with or without potholes by replacing the backbone feature extraction network in CNN and comparing the accuracy of different backbone networks in colour and colour grayscale frames in a cross-sectional manner. Bhatia et al. [32] developed a method to predict whether an input thermal image is a pothole or a non-pothole, demonstrating that using the residual network as the backbone network can improve the model detection rate applied in night-time and foggy weather environments. Fan et al. [33] experimentally evaluated 30 CNNs for road crack image classification, where Progressive neural architecture search (PNASNet) achieved the best balance between speed and accuracy. However, the image classification only presents the object image and does not detect the details of road damage in the image.

Semantic segmentation: To address the shortcomings of the image classification that only classifies images with or without road damage and to be able to detect road damage at the pixel level more intuitively through the network, Pereira et al. [34] used U-Net for a semantic segmentation method of road and pothole images. Their network structure is divided into two parts, encoder and decoder, for feature extraction, feature fusion, and result in prediction. Based on this, to design a more advanced semantic segmentation model to improve the detection rate, Fan et al. [35] proposed a novel semantic segmentation pothole detection method that used a spatial pyramid pooling module composed of tandem hollow convolutions to integrate spatial contextual information after enhancing the feature extraction process using a channel attention mechanism, which helped to detect multi-scale road potholes. To address the problem of difficult road crack detection, Zhang et al. [36] improved AD-Net’s cracked road detection performance by adding atrous convolution between the encoder and decoder and introducing depth supervision in the decoder stage. Fang et al. [37], on the other hand, improved the performance of AD-Net by configuring Transformer Block at the encoder layer, an external attention mechanism in the coding layer to enhance the feature representation capability and mitigate the impact of interference factors such as shadows, noise, etc., on the detection of road cracks.

Object detection: Object recognition mainly includes localization and classification of road damage, and the main problem is to improve the accuracy of object localization and classification. At the same time, the processing speed of the whole process needs to be improved for the real-time detection conditions required by ITS. Many researchers have previously tried to contribute to these aspects based on classical networks such as SSD [38], Faster-RCNN [39], YOLO Series [40,41,42], and EfficientDet [43]. Wang et al. [44] used Faster-RCNN as a detection framework and ResNet-152 as a feature extraction network, a proposed method to detect and classify road damage. However, the significant overall parameters of the network lead to slow processing speed and do not have multi-scale detection capability. Yebes et al. [45], also based on Faster-RCNN, utilized Resnet101 with a faster processing speed as a feature extraction network. However, even after relaxing the IOU index to 0.4 for evaluation, the accuracy reached 75% while only running at 5–6 fps. Ukhwah et al. [46] and Dharneeshkar et al. [47] trained based on the YOLOv3 detection framework for the dataset and tested on different pothole images achieving good accuracy. Gupta et al. [48] was based on SSD and RetinaNet as the detection framework, using ResNet34 and ResNet50 as feature extraction networks to propose a method for pothole localization from thermal images to solve the detection difficulties caused by unfavourable weather with low visibility.

However, most of these methods in Table 2 cannot localize and classify multiple classes of road damage objects. Most of the road images used for detection need to be taken vertically and close to the ground. Although they have good processing speed, their application to autonomous driving early warning systems is limited.

This paper presents a lightweight end-to-end road damage detection network that is designed to automatically, quickly, and efficiently detect and classify road damage.

## 3. Methodologies

**Figure 1:**
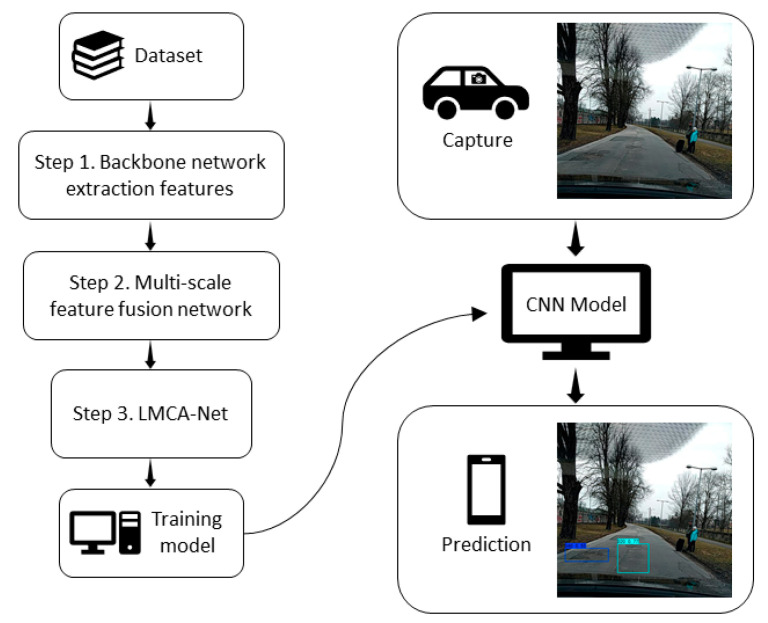
Flowchart of the road damage detection algorithm.

Figure 1 shows the flowchart of the proposed road damage detection algorithm, which firstly obtains a weight model by training a neural network, and, secondly, feeds the road images captured by vehicle cameras into the weight model to get prediction results.

**Figure 2:**
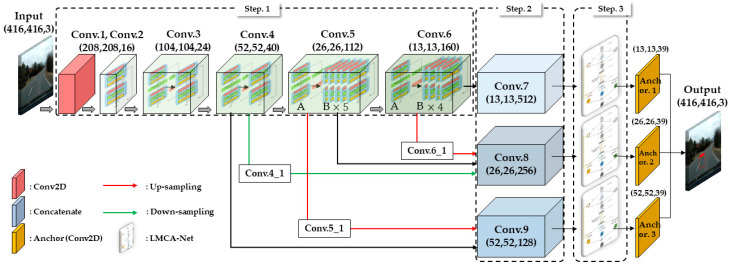
The network framework of the road damage algorithm is proposed in this paper.

The proposed network for road damage detection consists of three main steps, as shown in Figure 2: Backbone feature extraction network, Multiscale feature fusion network, and LMCA-Net.

In step 1, the Ghost Module was used as the basic module of the backbone feature extraction network to meet the overall lightweight of the network. In step 2, the diversity of features was enriched using the proposed multi-scale feature fusion network to improve the algorithm’s robustness in detecting different scales of damaged objects. In step 3, the finally obtained feature layers were fed into the lightweight multibranch channel attention network proposed in this paper. This embedded self-attentive module synthesizes feature information of different sizes with only a small number of operations.

### 3.1. Selection and Design of Backbone Network (Step 1)

For algorithmic systems designed for road damage detection, due to the limited memory and the computational resources of the application device, the efficiency and lightweight of the network itself are crucial, and how to make the network computationally less while ensuring accuracy is one of the main focuses of the research. Since the overall computation of a neural network depends mainly on the number of parameters of the backbone network, it is essential to choose a lightweight backbone network. For example, MobileNet [49] and ShuffleNet [50], with their deep convolution or channel-mixing operations, worked only on convolution and achieved lightness by small convolution kernels. As a backbone network, its primary role was to extract feature maps, and images could get many feature maps after passing through each convolution block. Still, many of these feature maps often had exceptionally high similarity and almost no variation. Such similar feature maps not only did not improve the network’s performance but also drove many convolutional layer calculations, consuming a lot of computational resources.

In contrast, GhostNet [51] conducted a different approach and obtained one of the similar feature maps by cheaply operating the transformation of another feature map so that one of the identical feature maps could be considered a phantom of the other. The phantom feature map could be generated by the cheap operations based on Ghost Module so that the same number of feature maps could be generated with fewer parameters than the ordinary convolutional layer, which required fewer arithmetic resources than the standard convolutional layer. In this paper, the Ghost module was chosen as the basis of the backbone network because it can improve the execution speed of the model in the neural network structure while ensuring efficient feature extraction.

**Figure 3:**
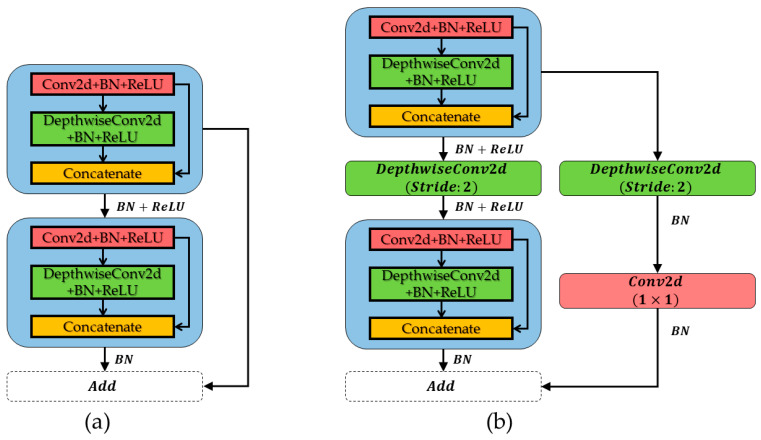
Two bottleneck structures based on ghost modules. (**a**) Bottleneck A, (**b**) bottleneck B.

Each feature block of the backbone network consists of two bottleneck structures, A and B, connected in series, as shown in Figure 3. Bottleneck A does not compress the height and width of the input feature layers and uses two Ghost modules for feature extraction and the residual to optimize the network. As for bottleneck B, depthwise convolution was added in the middle of the Ghost module and residual to compress the height and width of the input feature layer, respectively. The specific details are shown in Table 3.

The input image was passed into the backbone network based on the feature map obtained by one standard convolution. The feature map can be compressed and deepened by one Bottleneck B and multiple Bottleneck A according to the characteristics of the two bottleneck structures mentioned. We took the last three convolutional layers, namely Conv.4, Conv.5, and Conv.6, with shapes (52, 52, 40), (26, 26, 112), and (13, 13, 160), respectively. These three feature layers have multi-scale sensing feature information and contain three sizes that can be applied to objects near and far.

### 3.2. Multi-Scale Feature Fusion Network (Step 2)

Fusing features at different scales is an important way of improving detection performance. Shallow parts have higher resolution and contain more location and detail information, but they are less semantic and noisier. Deeper features have more robust semantic information but have low resolution and poor perception of details. The sizes of road damage detection targets vary, so the proposed multi-scale feature fusion network in this paper also integrates the feature layers into three dimensions of 13 × 13, 26 × 26, and 52 × 52 when fusing the shallow and deep layers.

The multi-scale feature fusion process is shown in Table 4, and the new feature layers (13, 13, 512) of Conv.7, (26, 26, 256) of Conv.8, and (52, 52, 128) of Conv.9 were finally obtained. Among them, Conv7 was directly obtained by expanding the number of channels of Conv6. Conv9 was generated by stacking Conv4 and upsampling Conv5. Conv8 was generated by stacking Conv5, downsampling Conv4, and upsampling Conv6. Such a feature fusion design can deepen the feature network and further enrich the diversity of features.

### 3.3. Lightweight Multibranch Channel Attention Network (Step 3)

**Figure 4:**
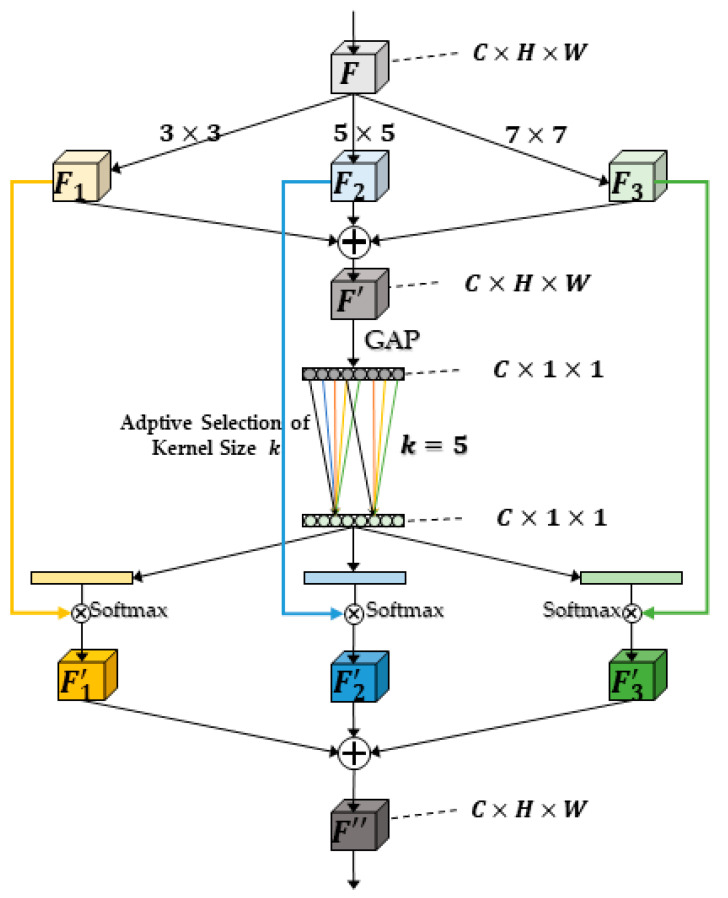
The overall structure of the LMCA-Net proposed in this paper.

Different sizes of the perceptual field of view, i.e., convolutional kernels, will have other effects on objects of different scales. Usually, attention mechanisms are often added to add weights to convolutional kernels to improve their ability to distinguish information during CNN design. Multi-scale convolutional kernels are critical to obtaining more feature information because of the different object sizes when performing road damage detection. In this paper, we propose a LMCA-Net, which aims to embed fewer attention modules in the network to improve detection efficiency. The overall structure is shown in Figure 4.

Firstly, the input feature map *F* was convolved with convolution kernels of size *3 × 3*, *5 × 5*, and *7 × 7* to obtain three feature maps, *F*_1_, *F*_2_, and *F*_3_, and then summed to obtain *F’* of shape *C × H × W* as in Equation (1).

(1) $$\begin{array}{c} F_{1}=D_{Conv}{}_{n^{3\times 3}}\left(F\right),\;F_{2}=D_{Conv}{}_{n^{5\times 5}}\left(F\right),\;F_{3}=D_{Conv}{}_{n^{7\times 7}}\left(F\right),\\ F^{\prime }=F_{1}+F_{2}+F_{3}, \end{array}$$

where *D_Conv_* is the dilated convolution and *n* is the convolution kernel size.

Next, the average pooling was performed along the *H* and *W* dimensions. Finally, a 1D vector of information about the feature dimension was obtained, with the shape of *C × 1 × 1* as in Equation (2).

(2) $$Avgpool\left(F^{\prime }\right)=\frac{1}{H\times W}\sum_{i=1}^{H}\sum_{j=1}^{W}F^{\prime }\left(i,j\right),$$

where *H*, *W* is the height and width of the input feature map, and (*i*, *j*) denotes the location of the feature points.

**Figure 5:**
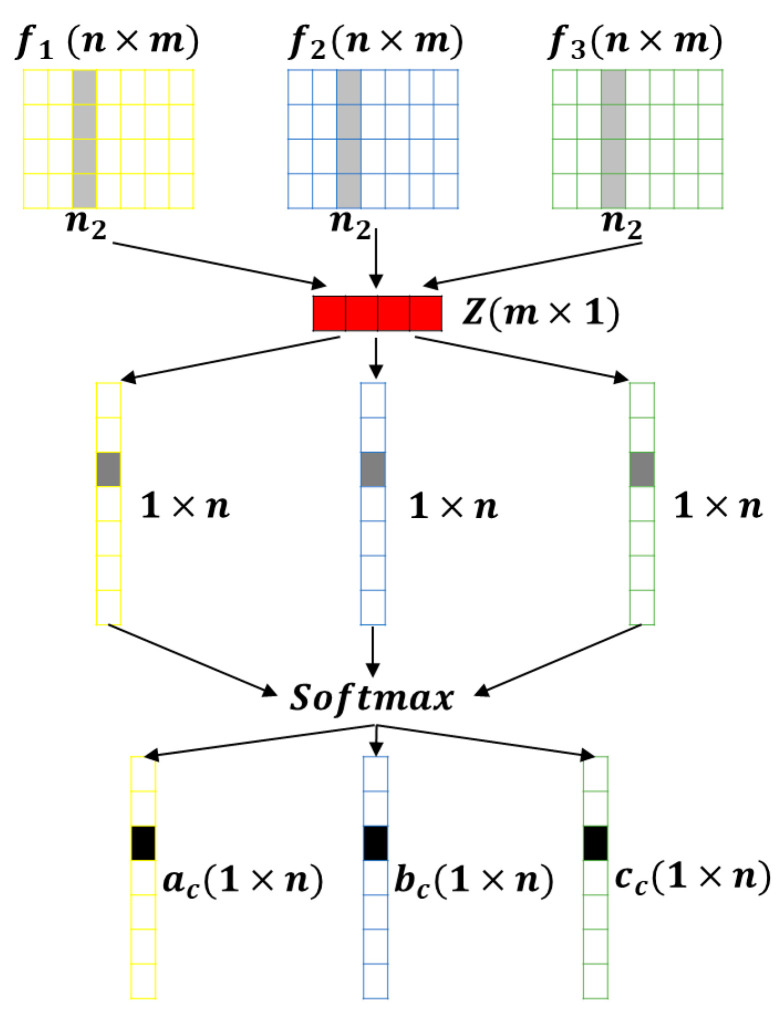
Information capture for cross-channel interactions in LMCA-Net with 1D convolution.

Such a vector can express the importance of the information of each channel. Next, a 1D convolution was used to map the original *C* dimension into *Z* dimension information. Following that, three 1D convolutions were used to change from the *Z* dimension to the original *C*. This completes the information extraction of the channel dimension. Compared with the fully connected layer in linear transform, 1D convolution can effectively capture the information of cross-channel interactions while significantly reducing the number of parameters [52]. Softmax was used for normalization. At this time, each channel corresponds to a score, representing the importance of its channel, which is equivalent to a mask, as shown in Figure 5.

Finally, the three separately obtained masks are multiplied by the corresponding *F*_1_, *F*_2_, and *F*_3_ to obtain *F*′_1_, *F*′_2_, and *F*′_3_. These three feature modules are summed and combined with the residuals of the original feature *F* for information fusion to obtain the final feature module *F*″ as in Equation (3), which has been refined compared with the original *F* and fused with information from multiple sensory fields.

(3) $$\begin{array}{c} F^{\prime \prime }=a_{c}\times F_{1}+b_{c}\times F_{2}+c_{c}\times F_{3}+F,\\ a_{c}+b_{c}+c_{c}=1, \end{array}$$

where *a_c_*, *b_c_*, and *c_c_* are the weights obtained after the Softmax function’s normalization, whose sum is 1.

## 4. Experiments and Discussion

### 4.1. Dataset and Experimental Environment

**Figure 6:**
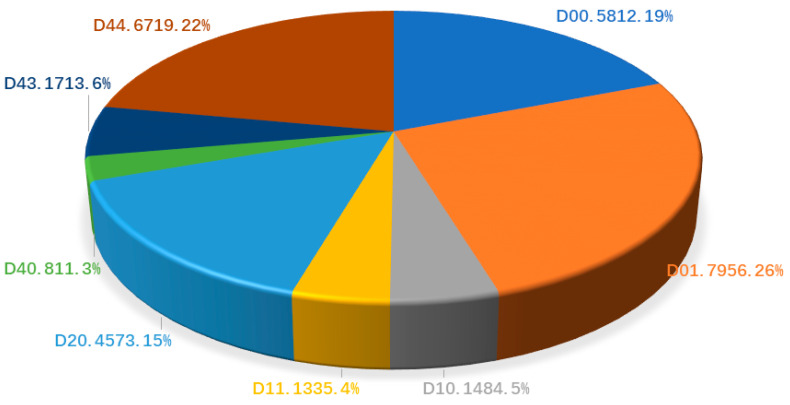
The number of each road damage categories in the GRDDC’2020 dataset as a percentage of the ground truth.

The road damage detection network proposed in this paper was evaluated using the Global Road Damage Detection Challenge (GRDDC’2020) dataset [53]. The dataset consists of 21,040 annotated images containing damage information collected from three countries, Japan, India, and the Czech Republic, with road damage information composed of the coordinates of bounding boxes and labels describing the type of damage associated with the boxes. We randomly divided the training and validation sets into 18,936 and 2104 images in a 9:1 ratio. In our experiments, a total of eight types of damage were selected as the detection objects to explore the detection efficiency of the proposed method for multiple types of road damage. Table 5 shows the specific road damage types and their definitions. Figure 6 shows the percentage of ground truth for each object in the dataset.

This paper’s experimental setup is summarized in Table 6. The experiment was built using the TensorFlow2 framework, and the results were computed using CUDA kernels. The hardware mainly consisted of a high-performance workstation host. The workstation was equipped with an Intel(R) Core (TM) i5-11400F processor and an RTX 3050 graphics card.

### 4.2. Evaluation Metrics and Experimental Details

To test the model’s performance, we used the following metrics to evaluate the model. The model was evaluated by introducing the average precision (AP) as in Equation (7), the mean average precision (mAP) as in Equation (8), and the F1 score as in Equation (6). The larger the value of these metrics, the higher the agreement of the prediction results with the ground truth.

The AP is calculated using the difference–average precision metric, the area under the precision–recall curve. The equations for precision and recall are shown in Equations (4) and (5).

(4) $$\mathrm{Precision}=\frac{\mathrm{TP}}{\mathrm{TP}+\mathrm{FP}},$$

(5) $$\mathrm{Recall}=\frac{\mathrm{TP}}{\mathrm{TP}+\mathrm{FN}},$$

(6) $$F1=2\times \frac{\mathrm{Precision}\times \mathrm{Recall}}{\mathrm{Precision}+\mathrm{Recall}},$$

where T/F denotes true/false, which indicates whether the prediction is correct, and P/N denotes positive/negative, which indicates a positive or negative prediction result.

(7) $$\mathrm{AP}=\frac{1}{n}\sum_{\left(r\in \frac{1}{n},\;\frac{2}{n}\;\ldots \;\frac{n-1}{n},\;1\right)}P_{\mathrm{interop}}\left(r\right),$$

(8) $$\mathrm{mAP}=\frac{1}{n}\sum \mathrm{AP},$$

where *n* denotes the number of detection points, and *P*_interop_ (*r*) represents the value of the accuracy at a recall of *r*.

**Figure 7:**
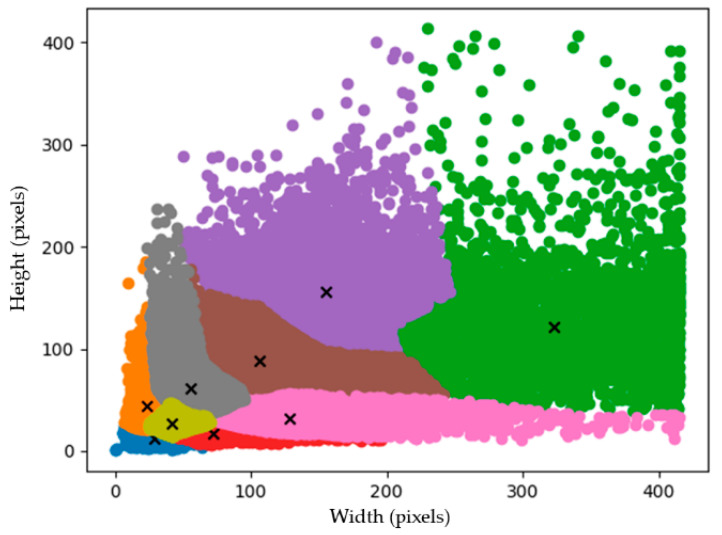
The clusters of height and width of the anchor boxes are derived by the K-means clustering.

The hyperparameters set in the training process are shown in Table 7, where the input image size is 416 × 416, the image batch size is 16, the overall training is 500 epochs, the maximum learning rate of the model is 0.01, and the minimum learning rate is 0.0001. Cosine annealing [54] was used as the learning rate descent method, CIoU [55] was used as the loss function, and mosaic and mix-up methods were used for data augmentation. Using anchor-based for prediction, a total of nine prior boxes were set for the three output feature layers, i.e., each feature layer had three different sizes of prior boxes for adjustment in prediction. The size of the anchor box was calculated by analysing the results of the dataset by the K-means clustering as in Figure 7, where the anchor mask is shown in Table 8.

### 4.3. Ablation Experiments

To validate the rationality and effectiveness of our proposed network, the effect of different backbone networks and module combinations on the results was further discussed in the ablation experiments. As a fair comparison, the required dataset for training, input image size, relevant hyperparameters, training strategy, and experimental environment were the same in the ablation experiments, except for the added module parameters.

First, we compared the backbone network in our model with several other classical backbone networks after replacement to evaluate the proposed approach. We selected six types of widely used representative deep neural networks and defined them as baseline models. Among them, Mobilenetv1 [49], Mobilenetv2 [56], and Mobilenetv3 [57] were lightweight backbone networks with faster processing speeds. VGG16 [58] featured a simple structure and was widely used as a feature extraction network for various CNN classical models. Resnet50 [59] had a deeper network that could achieve higher accuracy Densenet121 [60], on the other hand, achieved feature reuse and improved efficiency through the connection on the channel. However, VGG16, Resnet50, and Densenet121 all had many parameters, which were very computationally expensive. These baseline models were developed based on specific usage purposes, and all had good performance in prior studies, so we compared them with the approach proposed in this paper.

**Figure 8:**
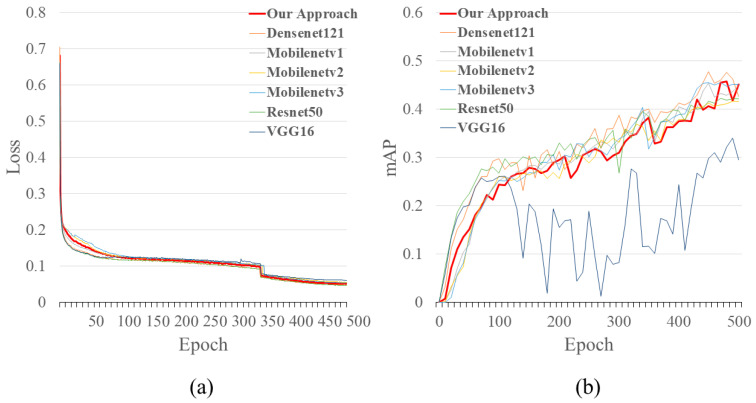
The upward trend of (**a**) loss function convergence status and (**b**) mAP for each model at 500 training epochs.

Figure 8 shows the trend of the loss function and mAP for each model when trained after 500 epochs. In Figure 8a, it can be seen that the proposed method’s loss function decreases to reach convergence when configuring different backbone networks, which proves the reasonableness of the network. It was also found that the proposed method converges faster than other backbone networks. On the other hand, in Figure 8b, except for VGG16, the mAP of all networks steadily increases with the iterative training of the network. It is worth noting that the mAP in Figure 8b is obtained from the validation set and the parameters set to speed up the evaluation are conservative, only to visualize the change in mAP during training, and the actual mAP for each model is shown in Table 9.

**Figure 9:**
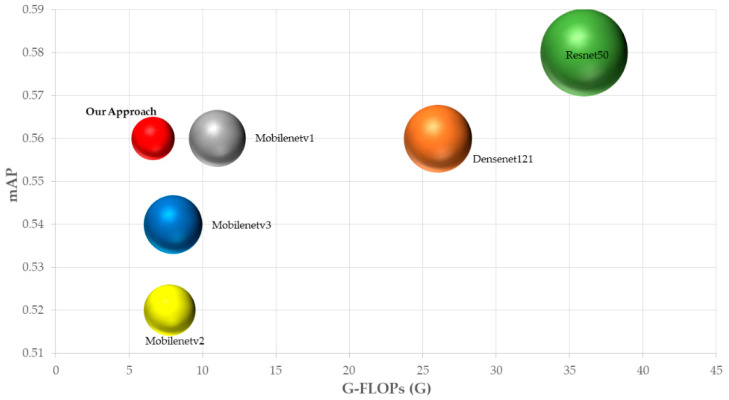
G-FLOPs vs. mAP. Details are in Table 9.

A discussion of the comparative results of the models in Table 9 shows that VGG16 has the lowest detection accuracy with a mAP of only 0.34 and the largest number of parameters. The remaining model with the highest combined accuracy is Resnet50, but it also has several parameters second only to VGG16. Our proposed method is similar to the MobileNet series in terms of combined accuracy, but our proposed model is less complex, with FLOPs of only 6.633 G, and for crosswalk blur (D43) and rutting, bump, pothole (D40), etc., the best detection results are achieved. Figure 9 shows a more intuitive and comprehensive comparison of the model performance. The horizontal coordinates represent the model’s complexity for evaluating the algorithm’s speed, the vertical coordinates represent the model’s comprehensive accuracy, and the sphere’s size represents the model’s number of parameters. Our proposed method is closer to the upper left than the other methods, and the performance is better than the other networks.

The effect of different module combinations on the results is additionally discussed. As shown in Table 10, when the original base algorithm (baseline) extracted features using only the backbone network and output results without adding any modules, the mAP was only 31.3%. After adding the multi-scale feature fusion network, the results improved to 47.8%. In addition, to discuss the performance of our proposed attention mechanism LMCA-Net, we selected four types of representative attention mechanisms and configured them each in step 3 of the network.

**Figure 10:**
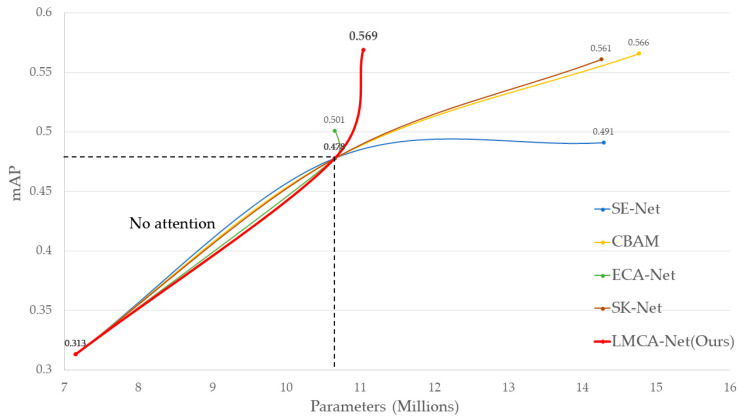
Performance on the baseline after adding each attention module.

As a fair comparison, LMCA-Net was evaluated on the same dataset, input image size, relevant hyperparameters, training strategy, and experimental setting by comparing four widely used methods. The experimental results are shown in Figure 10, where it can be seen that almost all methods improve detection accuracy. Still, due to the multi-scale perceptual field of our approach, the detection accuracy is higher for road damage objects of varying sizes compared to the channel attention SENet [61] and ECA-Net [62], which utilize a multilayer perceptron around learning correlations between channels. While the same level of accuracy is achieved with CBAM [63], which combines channel and spatial attention, and SK-Net [64] and performs engagement on convolutional kernels, our method has a smaller number of parameters and a stronger tendency for performance improvement.

**Figure 11:**
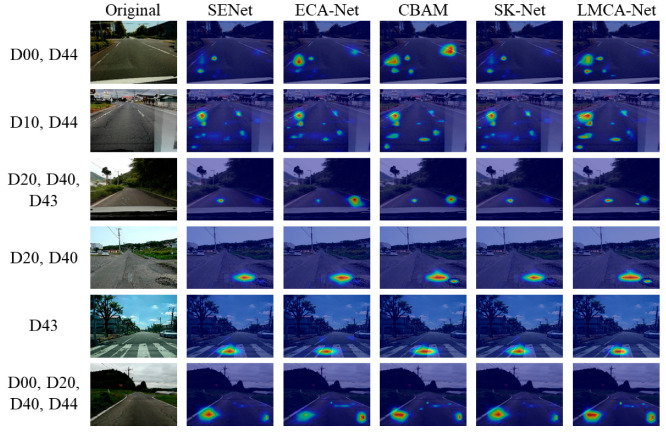
The visualized heat maps are generated by adding each attention mechanism.

As can be seen from the heat map shown in Figure 11, all methods achieve good attentional results due to the large size of the Crosswalk and white line blur objects. For longitudinal linear crack detection, our proposed LMCA-Net is slightly inferior to CBAM. For small object detection like bumps and potholes, LMCA-Net can ultimately achieve the same attention effect as CBAM and SK-Net. For the case of multiple objects combined, our method accurately generates more highlighted regions for multi-scale objects. It can achieve the same attention effect as the larger model with a smaller number of parameters.

Finally, we compared the road damage detection algorithm proposed in this paper with the widely used object detection algorithms SSD [39], Faster-RCNN [40], YOLO series [41,42,43], and EfficientDet [44]. The quantitative experimental results using the same dataset and model training methods are shown in Table 11.

**Figure 12:**
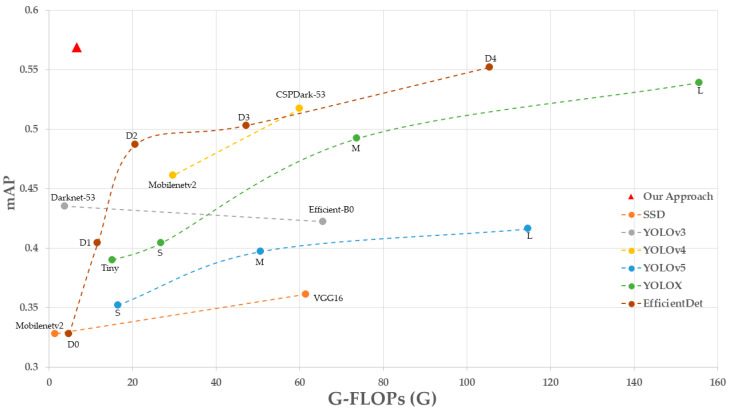
G-FLOPs(G) vs. mAP. Details are in Table 11. Note that our approach obtains higher accuracy while having less model complexity.

The models in the experiments were compared by replacing the backbone network, as shown in Figure 12. The accuracy of almost all the lightweight models was low compared to our proposed method because the overall network expression capability is insufficient to cover the detailed features of each detection target after replacing the smaller network. With the network structure’s complexity, each model’s accuracy increases, especially the accuracy of both EfficientDet-D4 and YOLOX-L, which is very high. Still, the number of parameters reaches 20.56 M and 54.15 M. While the approach suggested in this research is just 11.04 M in size, its model complexity and numerous parameters are substantially lower than those of previous methods with comparable accuracy, thanks to network design rationality and a light-weight attention mechanism. The lesser the model complexity, the less processing power required and the faster the prediction speed. The suggested model’s real-time processing speed is 31 frames per second, which is not the quickest when compared to other models but is sufficient to meet the demand for real-time detection.

**Figure 13:**
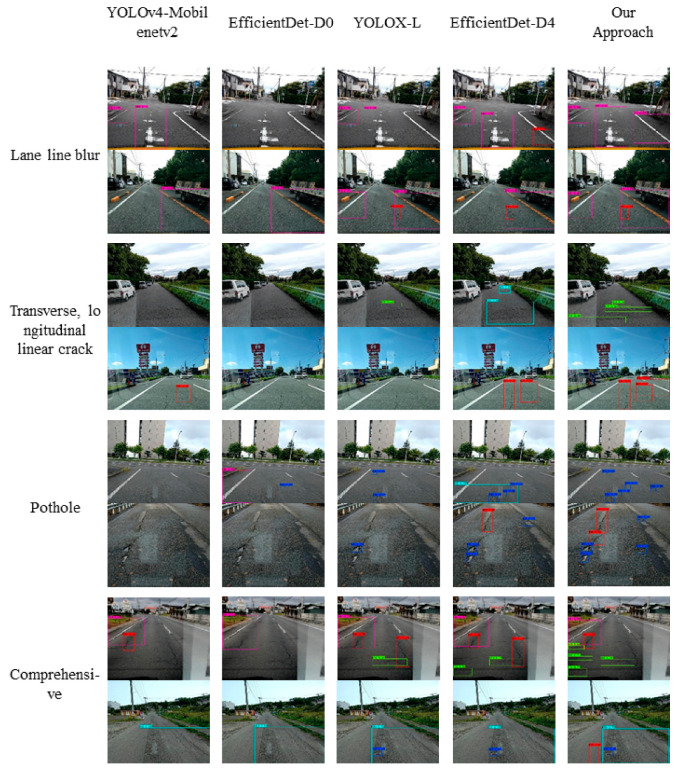
Comparison of detection samples of four representative models with the proposed method.

To evaluate the visualization results of the models, five representative models of YOLOv4-Mobilenetv2, EfficientDet-D0, YOLOX-L, EfficientDet-D4, and Our Approach of lightweight or accurate models are provided, as shown in Figure 13. These examples were taken from images of the test set covering the significant road damage, including transverse and longitudinal linear cracks, alligator cracks, bumps, potholes and crosswalks, and lane line blur. It can be seen that our proposed method outperforms the other models in terms of both classification and confidence scores. Among them, for the lightweight models YOLOv4-Mobilenetv2 and EfficientDet-D0, which have similar parameters, there are more deficiencies in pavement damage detection, such as cracks, potholes, etc. In comparison to the representative models YOLO-X and EfficientDet-D4, which have higher accuracy, our proposed method not only has absolute advantages in terms of the number of parameters, but it also performs better for the classification of small-sized targets like potholes and transverse linear cracks.

**Figure 14:**
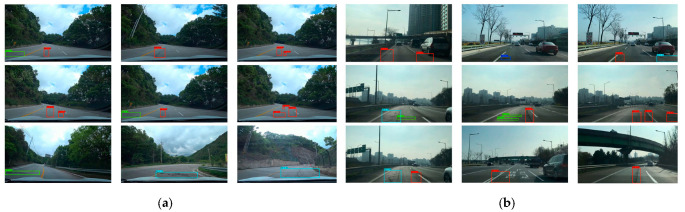
Examples of real-time detection of the proposed method cover (**a**) suburban and (**b**) urban road conditions in Korea.

Finally, to evaluate the performance of the proposed model on real roads outside the training dataset, it was again used to test the pixel size of 1920 × 1080 images obtained from Korean urban and suburban car recorders. Using the same non-maximum suppression method and setting a threshold fraction of 0.5 or higher to remove the excess boundary box, the video processing speed can reach about 30 fps. Due to the high-speed movement of the vehicle and bumps, the images obtained by the camera will appear to be inaccurately focused, thus making the target detection missing. However, the continuous frame detection results, as in Figure 14, are not difficult to find, even if there is a missed detection. However, a more comprehensive detection result can be achieved based on synthesizing multiple frames. In addition, detection is not only limited to the lane in which the video vehicle is traveling; adjacent lanes can also trigger prediction. These visualization results prove that our proposed model has comprehensive detection capability.

## 5. Conclusions

In this paper, we designed a lightweight end-to-end road damage detection network designed to quickly and automatically identify and classify specific types of road damage accurately. Such an efficient road damage detection method can reduce the risk of road damage to drivers and reduce the budget for road maintenance work. This study’s primary contributions are as follows. (1) The designed feature extraction and multi-scale fusion network, which is more advantageous for target recognition and classification at diverse distances and angles. (2) The proposed embedded lightweight attention module can improve detection accuracy with fewer parameters than previous studies by assigning weights to the multi-scale convolution kernel. The results of various ablation experiments evaluated for backbone networks, attention mechanisms, and other widely used target detection models show that our approach achieves significant performance improvements with few computations. The detection frame rate can be maintained at 30 fps when applied to real-world tests of high-definition road images. In contrast, continuous frames are capable of real-time detection and classification. However, the algorithm suffers from some limitations, such as false detection in shadow coverage, missed detection, and lack of exploration of detection at night or in low light. Overall, this work provides new ideas for existing road damage detection and models lightweight efforts. In the future, it is necessary to enrich the diversity of detection environments further and to explore the integration of road damage detection with other monitoring, warning, and tracking techniques.

## Figures and Tables

**Table 1:** Summary of road damage detection papers in traditional image processing.

Objective	Key Algorithm(s)	Reference
Pothole Detection	Histogram thresholds + Elliptical regression	Koch and Brilakis [21]
	Geometric features + Decision tree labelling	Schiopu et al. [22]
	RGB colour space	Jakštys et al. [23]
	Otsu + Boundary elimination	Akagic et al. [24]
	Inverse Binary + Otsu + Watershed	Chung et al. [28]
	LS-SVM	Hoang [29]
Crack Detection	Grayscale histograms + Otsu	Akagic et al. [25]
	Otsu + GLCM + SVM	Sari et al. [26]
	Modified Otsu	Quan et al. [27]
	LIBSVM	Gao et al. [30]

**Table 2:** Summary of road damage detection papers in deep learning.

Method	Objective	Key Algorithm(s)	Reference
Image Classification	Pothole	CNN	An et al. [31]
	Pothole	ResNet	Bhatia et al. [32]
	Crack	PNASNet	Fan et al. [33]
Semantic Segmentation	Pothole	U-Net	Pereira et al. [34]
	Pothole	SPP + Channel attention	Fan et al. [35]
	Crack	AD-Net	Zhang et al. [36]
	Crack	Transformer Block + AD-Net	Fang et al. [37]
Object Detection	Crack	ResNet-152 + Faster-RCNN	Wang et al. [44]
	Crack	Resnet101 + Faster-RCNN	Yebes et al. [45]
	Pothole	YOLOv3	Ukhwah et al. [46]
	Pothole	YOLOv3	Dharneeshkar et al. [47]
	Pothole	SSD + RetinaNet	Gupta et al. [48]

**Table 3:** Details of the processes in Step 1.

Blocks	Layer	Output Shape	Parameters	Total Parameters
Image	Input	416 × 416 × 3	0	2,408,184
Conv.1	Conv2d + BN + Leaky	208 × 208 × 16	496	
Conv.2	Bottleneck A × 1,	208 × 208 × 16	528	
Conv.3	Bottleneck A × 1, Bottleneck B × 1	104 × 104 × 24	2884	
Conv.4	Bottleneck A × 1, Bottleneck B × 1	52 × 52 × 40	12,496	
Conv.5	Bottleneck A × 1, Bottleneck B × 5	26 × 26 × 112	448,908	
Conv.6	Bottleneck A × 1, Bottleneck B × 4	13 × 13 × 160	1,942,872	

**Table 4:** Details of the processes in Step 2.

Blocks	Layer	Output Shape	Parameters	Total Parameters
Conv.6	Input	13 × 13 × 160	0	1,516,928
Conv.7	Conv2d + BN + Leaky, Depthwise_Conv2d + BN + Leaky, Conv2d + BN + Leaky, Conv2d + BN + Leaky	13 × 13 × 512, 13 × 13 × 512, 13 × 13 × 1024, 13 × 13 × 512	1,145,344	
Conv.6_1	Conv2d + BN + Leaky, Up_Sampling2D	26 × 26 × 256	132,096	
Conv.5	Input, Conv2d + BN + Leaky	26 × 26 × 112, 26 × 26 × 256	29,696	
Conv.4	Input	52 × 52 × 40	0	
Conv.4_1	Conv2d + BN + Leaky, Down_Sampling2D	26 × 26 × 256	135,424	
Conv.8	Concatenate (Conv.6_1, Conv.5, Conv.4_1)	26 × 26 × 256	0	
Conv.5_1	Conv2d + BN + Leaky, Up_Sampling2D	52 × 52 × 128	68,736	
Conv.4	Conv2d + BN + Leaky	52 × 52 × 128	5632	
Conv.9	Concatenate (Conv.5_1, Conv.4)	52 × 52 × 128	0	

**Table 5:** Eight categories of road damage and their definitions in the GRDDC’2020 dataset.

Class Name	Damage Detail	Damage Type	
D00	Tire indentation	Longitudinal linear crack	Linear crack
D01	Construction joint		
D10	Equal interval	Transverse linear crack	
D11	Construction joint		
D20	Partial or overall pavement	Alligator crack	
D40	Rutting, bump, pothole, separation	Other corruption	
D43	Crosswalk blur		
D44	lane line blur		

**Table 6:** Workstation mainframe hardware and software.

Items		Description
H/W	CPU	Intel(R) Core (TM) i5-11400F
	RAM	16 GB
	SSD	Samsung SSD 500GB
	Graphics Card	NVIDIA GeForce RTX 3050
S/W	Operating System	Windows 11 Pro, 64bit
	Programming Language	Python 3.7
	Learning Framework	TensorFlow 2.2.0

**Table 7:** The hyperparameters set during the training of the proposed method.

Input Settings			Loss Calculation					Data Enhancement	
Input shape	Batch size	Total Epoch	Loss function	Anchor-based	Max_lr	Min_lr	Decay type	Mosaic	Mixup
416 × 416	16	500	CIoU	True	0.01	0.0001	Cosine Annealing	True	True

**Table 8:** The anchor mask setting parameters derived from Figure 7.

Anchor Layer	Anchor Size (Width, Height)
Anchor. 1	(29, 11); (23, 43); (42, 27)
Anchor. 2	(72, 16); (55, 61); (128, 31)
Anchor. 3	(106, 88); (155, 156); (322, 121)

**Table 9:** Comparison of quantitative experimental results on the same test set, where bold numbers indicate the best value for each column, where the Precision and Recall calculated in this paper represent when the threshold value is 0.5.

		MobileNetv1	MobileNetv2	MobileNetv3	VGG16	ResNet50	DenseNet121	Our Approach
Precision	D00	0.66	0.76	0.70	0.69	0.67	0.64	0.76
	D01	0.98	0.99	0.00	1.00	0.00	0.00	1.00
	D10	0.70	0.83	0.65	1.00	0.63	0.72	0.80
	D11	0.99	0.99	1.00	0.00	1.00	1.00	1.00
	D20	0.75	0.82	0.80	0.82	0.76	0.74	0.79
	D40	0.73	0.79	0.70	0.71	0.74	0.80	0.89
	D43	0.89	0.92	0.93	0.80	0.93	0.96	0.96
	D44	0.73	0.75	0.75	0.82	0.72	0.75	0.75
Recall	D00	0.19	0.14	0.17	0.06	0.22	0.22	0.15
	D01	0.04	0.04	0.00	0.04	0.00	0.00	0.04
	D10	0.08	0.05	0.06	0.01	0.20	0.14	0.05
	D11	0.33	0.33	0.33	0.00	0.33	0.33	0.33
	D20	0.51	0.49	0.50	0.34	0.53	0.56	0.47
	D40	0.27	0.20	0.19	0.18	0.31	0.32	0.37
	D43	0.71	0.68	0.69	0.55	0.72	0.69	0.81
	D44	0.51	0.48	0.45	0.25	0.57	0.53	0.49
F1	D00	0.30	0.24	0.28	0.12	0.33	0.33	0.25
	D01	0.08	0.08	0.00	0.08	0.00	0.00	0.08
	D10	0.15	0.10	0.12	0.01	0.31	0.24	0.10
	D11	0.50	0.50	0.50	0.00	0.50	0.50	0.50
	D20	0.61	0.61	0.61	0.48	0.63	0.64	0.59
	D40	0.39	0.32	0.30	0.29	0.44	0.46	0.32
	D43	0.79	0.78	0.80	0.65	0.81	0.80	0.85
	D44	0.60	0.58	0.56	0.39	0.64	0.62	0.59
AP	D00	0.34	0.36	0.36	0.27	0.36	0.37	0.35
	D01	0.50	0.32	0.36	0.16	0.45	0.31	0.51
	D10	0.30	0.35	0.30	0.19	0.34	0.37	0.31
	D11	0.83	0.67	0.92	0.00	0.92	0.81	0.63
	D20	0.60	0.64	0.61	0.53	0.62	0.62	0.61
	D40	0.44	0.42	0.39	0.33	0.49	0.50	0.40
	D43	0.81	0.80	0.78	0.69	0.81	0.82	0.91
	D44	0.65	0.65	0.63	0.56	0.66	0.66	0.73
mAP		0.56	0.52	0.54	0.34	0.58	0.56	0.57
G-FLOPs (G)		10.129	7.763	7.178	111.845	35.105	26.050	6.633
Parameters (Millions)		12.304	11.413	13.341	23.550	33.293	18.051	**11.041**

**Table 10:** The results of the ablation experiments using the attention module of the same dataset.

Backbone Feature Extraction	Baseline	√	√	√	√	√	√
Multi-scale feature fusion		√	√	√	√	√	√
SE-Net			√				
CBAM				√			
ECA-Net					√		
SK-Net						√	
LMCA-Net (Ours)							√
Parameters (Millions)	7.149	10.657	14.290	14.771	10.657	14.258	11.041
mAP	0.313	0.478	0.491	0.566	0.501	0.561	0.569
The “√” in each column indicates that the leftmost component is used in the model.							

**Table 11:** Comparison of the proposed method with SSD, Faster-RCNN, YOLO series, and EfficientDet on the same dataset.

Method		Input Size	Backbone	Parameters (Millions)	FPS	G-FLOPs (G)	mAP (%)
SSD		300 × 300	VGG16	24.54	29	61.45	0.361
		300 × 300	Mobilenetv2	4.47	35	1.53	0.328
YOLOv3		416 × 416	Darknet-53	61.56	27	65.65	0.422
		416 × 416	Efficient-B0	7.02	21	3.84	0.435
YOLOv4		416 × 416	CSPDark-53	63.98	19	60.01	0.517
		416 × 416	Mobilenetv2	39.06	28	29.74	0.461
YOLOv5	S	640 × 640	CSPDarknet53 + SPP	7.08	36	16.54	0.352
	M	640 × 640	CSPDarknet53 + SPP	21.09	23	50.69	0.397
	L	640 × 640	CSPDarknet53 + SPP	46.67	15	114.68	0.416
YOLOX	Tiny	640 × 640	Modified CSP	5.03	31	15.24	0.390
	S	640 × 640	Modified CSP	8.94	31	26.77	0.404
	M	640 × 640	Modified CSP	25.29	20	73.75	0.492
	L	640 × 640	Modified CSP	54.15	14	155.70	0.539
Faster-RCNN		600 × 600	VGG16	136.83	8	369.89	0.475
		600 × 600	ResNet50	28.35	7	941.01	0.499
EfficientDet	D0	512 × 512	Efficient-B0	3.83	13	4.78	0.328
	D1	640 × 640	Efficient-B1	6.56	10	11.59	0.404
	D2	768 × 768	Efficient-B2	8.01	9	20.71	0.487
	D3	896 × 896	Efficient-B3	11.91	7	47.23	0.503
	D4	1024 × 1024	Efficient-B4	20.56	4	105.55	0.552
Our Approach		416 × 416	Ghost module	11.04	31	6.63	0.569

## Data Availability

Not applicable.
